# Knowledge of, attitudes towards, and practice relating to schistosomiasis in two subtypes of a mountainous region of the People’s Republic of China

**DOI:** 10.1186/2049-9957-3-16

**Published:** 2014-05-07

**Authors:** Lu Liu, Guo-Jing Yang, Hong-Ru Zhu, Kun Yang, Lin Ai

**Affiliations:** 1 Key Laboratory of Parasitic Disease Control and Prevention (Ministry of Health), Jiangsu Provincial Key Laboratory of Parasite Molecular Biology, Wuxi, Jiangsu Province 214064, People’s Republic of China; 2 National Institute of Parasitic Diseases, Chinese Center for Disease Control and Prevention, and Key Laboratory on Biology of Parasite and Vector, Ministry of Health, and WHO Collaborating Center for Malaria, Schistosomiasis and Filariasis, Shanghai, People’s Republic of China

**Keywords:** Schistosomiasis, Knowledge, Attitude, Practice, Eryuan, P.R. China

## Abstract

**Background:**

Schistosomiasis japonica is still endemic in the People’s Republic of China (P.R. China) in five provinces of lake and marshland regions and in two provinces of mountainous regions. Studies elucidated that individual and community perception, attitudes towards schistosomiasis, and hygiene behaviors were crucial factors for preventing schistosomiasis. This study sought to assess the knowledge of, attitudes towards, and practices (KAP) relating to schistosomiasis in two subtypes of a mountainous region in Eryuan County, Yunnan Province, P.R. China. The study’s aim is to make suggestions for establishing more specific and effective control measures for disease transmission and interruption in two subtypes of a mountainous region with low-level infection rates.

**Methods:**

A cross-sectional study of 3,000 inhabitants was carried out in the Yongle (plateau basin) and Xinzhuang (plateau canyon) communities of Eryuan County, Yunnan Province in November and December 2011. Stratified cluster random sampling was undertaken using a uniform set of quantitative questionnaires administered by trained assistants. This was further supported with qualitative data from in-depth interviews (IDIs) conducted with ten farmers and ten students. All participants were examined for schistosomiasis using both a serological test (indirect hemagglutination assay [IHA]) and a stool examination (Kato-Katz).

**Results:**

The total schistosomiasis knowledge rate in Yongle (83.4%) was significantly lower than that in Xinzhuang (95.5%). In both communities, among the respondents aged 15 years or below, more than one third didn’t know the name, endemic areas, and animal reservoirs of schistosomiasis. The majority of respondents in Eryuan acquired their schistosomiasis knowledge from doctors, followed by handouts and hearing from others. The infection rate was once the highest in Yongle, but is now the highest in Xinzhuang, where there are more risk factors for schistosomiasis, such as frequently grazing cattle, digging vegetables or cutting grass in the field, as well as raising cattle by free grazing.

**Conclusion:**

In short, Eryuan County’s overall knowledge rate of schistosomiasis was found to be high. Due to various dominating risk factors, different control strategies should be designed keeping in mind the two different subtypes of endemic areas for schistosomiasis in mountainous regions, namely plateau basins and plateau canyons.

## Multilingual abstracts

Please see Additional file 1 for translations of the abstract into the six official working languages of the United Nations.

## Background

Schistosomiasis japonica, one of the neglected tropical diseases (NTDs) listed by the World Health Organization (WHO), has a documented history of 2,100 years in the People’s Republic of China (P.R. China) [1]. About 60 years ago, it was estimated that 11.6 million people living in 12 provinces of P.R. China had a huge burden of disease caused by schistosomiasis [2]. After the integrated strategy emphasizing snail elimination and synchronous chemotherapy for human and domestic animals, a new strategy of schistosomiasis control was established by the State Council of P.R. China at the beginning of the 21st century. This strategy was based on integrated measures emphasizing control of the infection source [3,4]. The number of schistosomiasis patients decreased from nearly 800,000 in 2000 to less than 287,000 in 2011, a drop of more than 60%. Moreover, the infection rates of human and livestock have both dropped below 5% (one of the criterion of transmission control in P.R. China) in 454 counties across the country [5]. At present, schistosomiasis is endemic in five provinces of the lake and marshland regions and two provinces of mountainous regions [6]. At this new stage of schistosomiasis control, China faces the challenge of keeping infection rates low and further interrupting the transmission of schistosomiasis [7].

Eryuan County is one of the mountainous endemic areas for *Schistosoma japonicum* in the Yunnan Province, where schistosomiasis infection rates dropped below 1% in 2009, with the aim to interrupt transmission by 2015 [8]. Although there are fewer snail-infested areas and patients in mountainous regions than that in other subtypes of endemic areas (plain river network regions and lake and marshland regions), transmission could easily reemerge due to various factors, such as a rough terrain, an unstable economy, and unreliable transportation [9]. For instance, resurgences were seen in 33.3% (4/12) of the counties where transmission of schistosomiasis had been interrupted and in 100% (3/3) of the counties already under control [1,10,11].

Recent studies support that both individual and community perception of, and attitudes towards, schistosomiasis, as well as its prevention and treatment, are important factors which influence transmission/infection rates [12]. Clean water and adequate sanitation, and changing hygiene behaviors are more effective key factors for sustainable control than snail elimination and synchronous chemotherapy [12,13].

In China, education programs have been implemented intensively [14,15], but even though a substantial number of knowledge, attitudes, and practices (KAP) studies being done in lake and marshland regions, few English-language articles have reported on KAP studies in mountainous regions in recent years. A handful of articles in Chinese on schistosomiasis in the Yunnan Province were confined to parts of the endemic areas, ignoring the different subtypes of the endemic mountainous areas [9,16]. Early reports suggest that different control strategies should be designed keeping in mind the environmental situation and socioeconomic factors in the two subtypes of endemic regions, i.e., plateau basins and plateau canyons [17]. This article describes the KAP of populations in two subtypes of the endemic areas in Eryuan County as a basis for the design and implementation of more specific and effective disease elimination strategies in mountainous regions with low-level infection rates.

## Methods

### Study site

**Figure 1:**
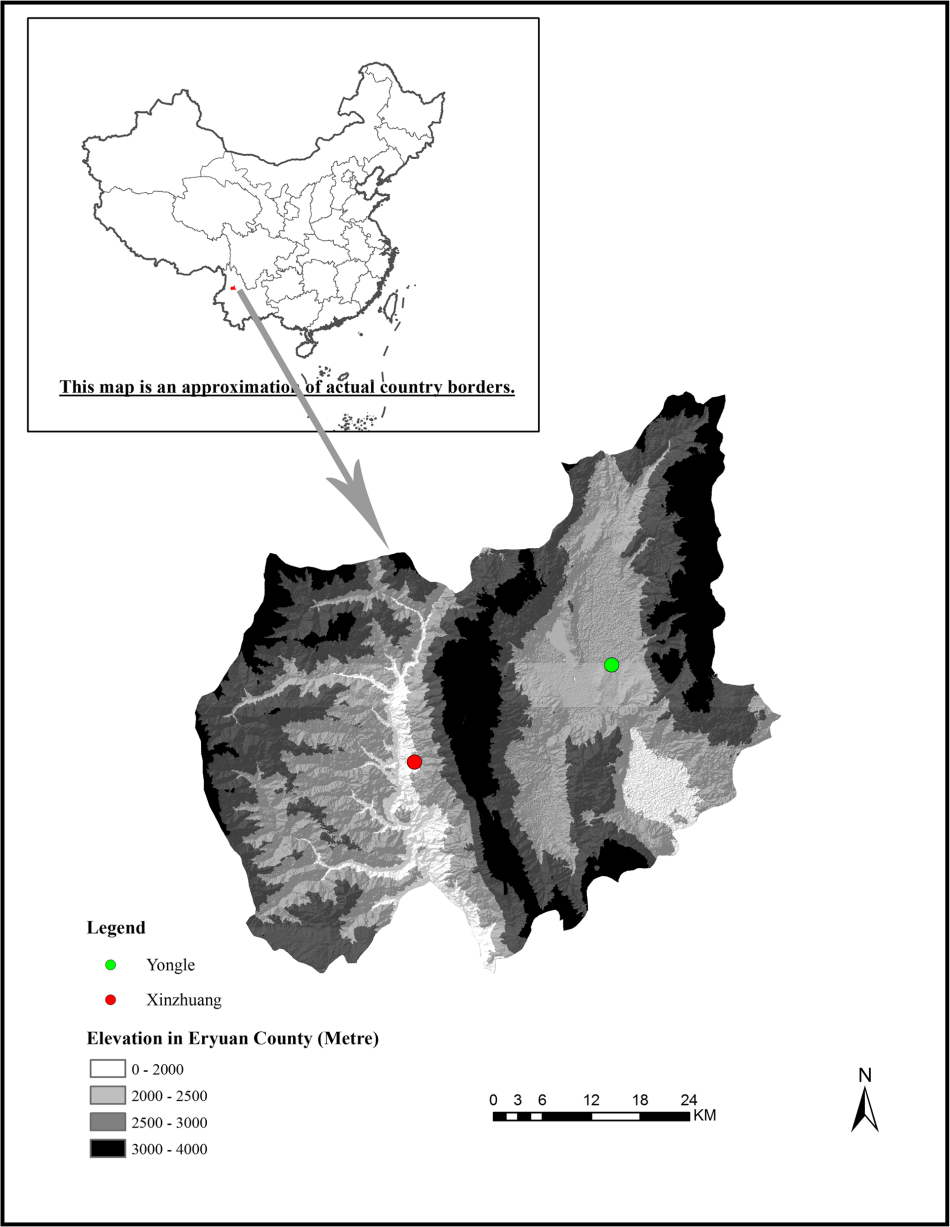
Two study sites in Eryuan County, Yunnan Province, People’s Republic of China.

The study was conducted in Eryuan County, Yunnan Province (99.54°–100.34°E, 25.80°–26.43° N) in November and December 2011. There are 300,000 people living in Eryuan, which has a total area of 2,961 km^2^. Bai and Han are the most important ethnic groups [16]. The majority of their members work in agriculture and/or animal husbandry. Eryuan has been endemic for schistosomiasis for at least 70 years. Two subtypes of mountainous endemic areas for *S. japonicum* exist: basin areas with an elevation of about 2,200 m and a canyon region below 2,000 m but with a peak of 3,000 m around it (see Figure 1) [18]. *Oncomelania hupensis* is distributed through irrigation canals and ditches in the basin areas while in the canyon areas, it is distributed as a single point or line in terraced fields [19]. The Yongle and Xinzhuang communities were purposively selected from the plateau basin areas and plateau canyon areas, respectively, because these two communities: (a) represent two different natural environments of typical mountainous areas; (b) had a reduction of the infection rate to 0.15% in 2009, the positive rate by a stool test is below 1%, and snail-infested areas have decreased by 98% compared to the largest area in the history [20]; and (c) had health authorities and residents who were willing to participate in this study.

### Study population

This was a cross-sectional study mainly based on quantitative questionnaires and partly supported by in-depth interviews (IDI) with local residents. Firstly, households were randomly selected from both communities with a percentage of 40%, i.e. 646 households in Yongle and 340 in Xinzhuang. A total of 3,000 residents aged ≥ five years in selected households (2,146 in Yongle and 854 in Xinzhuang) participated in the study excluding the family members who were not at home during the course of the study. Set α = 0.05 and β = 0.1; the number of the sample size was determined to be at least 780 in order to compare the knowledge rates of the two communities according to pretests and references. That is, the statistical power (1-β) in this study was above 0.9, which is above the recommended minimum value of 0.8. In addition, ten farmers and ten students (out of a total of 2,149 farmers and 550 students) were randomly selected from the members of community who were not involved in the study for IDIs, which were needed to provide some contextual meaning to the quantitative survey. Participating villagers underwent serological and parasitological tests so that the infection situation of *S. japonicum* could be determined.

### Data collection and analysis

A standard questionnaire was developed consisting of five parts: the social demographic status, medical records pertaining to the infection, schistosomiasis related knowledge, attitudes towards schistosomiasis and practice of anti-schistosomiasis measures. Most questions were single-choice questions except for the question asking respondents where and how they acquired knowledge about schistosomiasis. The questionnaire was first designed by experts in English and then translated into Chinese. Besides expert evaluation, pretesting in one non-selected community was done to assess content validity and appropriateness, and question comprehensibility. If necessary, the questionnaire would have been revised. In addition, 20 more villagers were given the questionnaire and were retested two weeks later without any educational intervention. The Cronbach’s alpha coefficient of internal consistency was used to estimate the reliability of the questionnaire. The alpha coefficient of the questionnaire was 0.75, which is above the recommended minimum value of 0.7.

Data collectors were trained for two days on how to conduct the interview. Individuals completed the questionnaire by themselves, while those who were illiterate or semiliterate were helped by the data collectors. Incomplete questionnaires were returned and participating households were revisited so that those questionnaires could be completed. Finally, 5% of the interviewed individuals were randomly selected and re-interviewed by the first author.

A venous blood sample of 3–5 ml and a stool sample from each respondent (modifier Kato-Katz thick smear, one stool sample for three slides) were collected by field workers and sent to the local schistosomiasis control station in Eryuan. According to the instructions of the *Handbook of Prevention and Cure of Schistosomiasis*[21], the serum samples were kept frozen and tested by indirect hemagglutination assay (IHA) for IgG antibodies against *S. japonicum*. Kato-Katz thick smears of 41.7 mg were prepared from the stool samples and read by experienced technicians. The number of eggs of each parasite species was counted and recorded separately. A sub-sample of 5% of the slides were reexamined by a senior technician for quality control.

Data were double entered into EpiData 3.0 by two different researchers and cross-checked for accuracy by the first author. Then, data analysis was performed using the Statistical Package for Social Science 18 (IBM SPSS Statistics, IBM Company, USA). Disparities of proportions were tested for statistical significance with a Chi-square test. A *P*-value <0.05 was considered significant. Descriptive and univariate analyses were conducted. A Chi-square test and binary logistic regression models (forward logistic regression with *P*-value <0.05 as inclusion criteria and *P*-value > 0.1 as exclusion criteria) were applied to analyze the relationship between the variables. In the knowledge part of the questionnaire, a correct answer was awarded one point and an incorrect answer or a “do not know” response was awarded zero points (the question relating to the source of schistosomiasis knowledge did not count). All points were then added up to get the total knowledge score for each respondent. The knowledge score ranged from zero to six. Previous or recent infection with *S. japonicum* was recorded according to the result of the serological and stool tests.

## Results

### Demographic characteristics of the study population

A total of 2,734 valid questionnaires (1956 [71.5%] in Yongle and 778 [28.5%] in Xinzhuang) were collected, with an overall response rate of 91% (see Table 1). These two communities showed no difference regarding immigrants. As shown in Table 1, there was a slightly higher number of females in both communities, with a significant difference between the genders. There were also significant differences in the categories of age, ethnicity, occupation, and education level. In the Yongle and Xinzhuang communities, the majority of the residents were farmers (76.8% and 83%, respectively), followed by students (21.8% and 15.8%, respectively). Primary-educated residents accounted for 48.2% and 50.6%, respectively; 36.6% and 28.9%, respectively, completed junior high school; and 10.6% and 16.3%, respectively, were illiterate or semiliterate (i.e. those who didn’t attend or finish primary school, or those who could not read or just knew a few words). Notably, the majority of residents (95.7%) in Yongle were of the Han nationality whereas residents of the Bai nationality (83.2%) were predominantly in Xinzhuang.

**Table 1:** Demographic characteristics of the study participants

**Characteristics**		**Total N = 2734**	**Community**		***χ***^**2**^	***P*-Value**
			**Yongle**	**Xinzhuang**		
			**N = 1956**	**N = 778**		
Gender	Male	47	48.3	43.7	4.647	**0.031**
	Female	53	51.7	56.3		
Immigrant	Yes	1.2	1.2	1.0	0.190	0.663
	No	98.8	98.8	99		
Age group (years)	5 – 15	20	21.7	15.7	45.64	**<0.001**
	16 – 30	19.3	21.4	14.3		
	31 – 45	34.3	33.2	37		
	46 – 65	25.4	22.9	31.7		
	≥66	1.0	0.8	1.3		
Ethnicity	Han	73.2	95.7	16.6	N/A	**<0.001**^**a**^
	Bai	26.4	3.8	83.2		
	Other	0.4	0.5	0.2		
Occupation	Farmer	78.6	76.8	83	N/A	**<0.001**^**a**^
	Fisherman	0.4	0.5	0.1		
	Herdsman	0.2	0.3	0		
	Worker	0	0.1	0		
	Student	20.1	21.8	15.8		
	Pre-school	0.3	0.4	0.1		
	Business	0.2	0	0.8		
	Government	0.1	0.1	0		
	Other	0.1	0.1	0.1		
Education	Illiterate	12.2	10.6	16.3	N/A	**<0.001**^**a**^
	Primary school	48.9	48.2	50.6		
	Junior high school	34.4	36.6	28.9		
	Senior high school	3.9	4.1	3.3		
	College and above	0.6	0.5	0.8		

All values are percentages.

N/A: not applicable.

^a^*P*-value based on Fisher’s exact test and *P*-value highlighted in bold means the difference was statistically significant.

### Medical records of the study population and current prevalence of *S. japonicum*

As elucidated in Table 2, a total of 1,078 (39.4%) respondents indicated an infection history of *S. japonicum*. The amount of respondents with an infection history of schistosomiasis in Yongle was more than those in Xinzhuang, in terms of the type and treatment frequencies. According to the parasitological and serological survey, the schistosomiasis seroprevalence in total was 12.8% (350/2734) and *S. japonicum* eggs were found in 21 (0.8%) respondents. In Yongle, 15% of the subjects were IHA-positive, which was a higher rate than in Xinzhuang (7.5%), with a significant difference. On the contrary, 0.3% of Kato-Katz tests were positive in Yongle, which was lower than that in Xinzhuang (1.4%). Among the respondents who were infected with schistosomiasis, people aged 31–45 accounted for the biggest proportion of Kato-Katz and IHA-positive subjects in both communities. In both communities, over 75% of Kato-Katz and IHA positive respondents were farmers.

**Table 2:** Medical records of the study population and the current prevalence

**Medical record**		**Total**	**Yongle**	**Xinzhuang**	***χ***^**2**^	***P*-Value**
		**N = 2734**	**N = 1956**	**N = 778**		
Infected by schistosoma	Yes	39.4	42.1	32.6	22.6	**<0.001**
	No	59.7	56.9	66.7		
	Unclear	0.9	1.0	0.6		
Schistosomiasis type	Chronic	86.4	83.9	94.5	23.8	**<0.001**
	Acute	12.6	15.4	4		
	Advanced	0.9	0.7	1.6		
Treatment frequencies	0 times	0.8	0.6	1.3	75.4	**<0.001**
	1 – 3 times	39.4	32.8	62.1		
	4 – 6 times	17.2	17.4	16.4		
	7 times	42.7	49	20.2		
Treatment based on	Stool positive	33.5	29.3	47.7	83.6	**<0.001**
	Blood positive	54.6	62.2	28.7		
	Other	11.9	8.4	23.6		
IHA	Positive	12.8	15.0	7.5	28.2	**<0.001**
Kato-Katz	Positive	0.8	0.3	1.4	8.14	**0.004**

Note: *P*-value highlighted in bold means the difference was statistically significant.

All values are percentages.

### Knowledge of, attitudes towards, and practice relating to schistosomiasis

**Figure 2:**
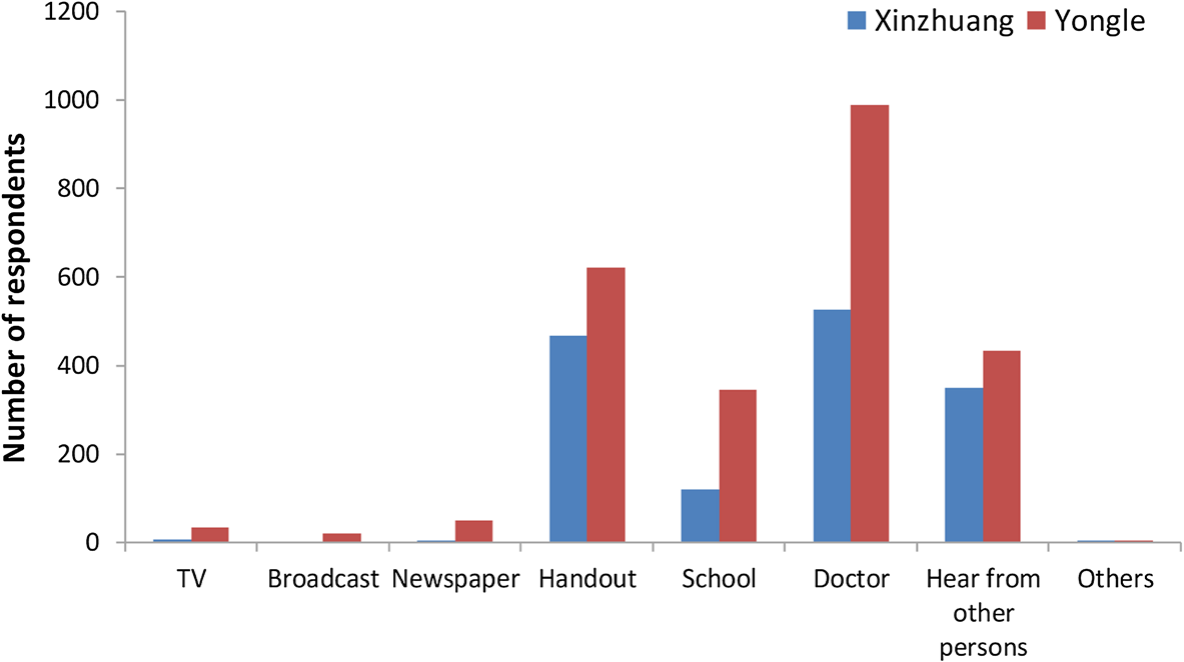
Sources of the respondents’ schistosomiasis-related knowledge.

The respondents’ knowledge of schistosomiasis is summarized in Table 3. Calculating the knowledge scores in the two communities (K1–K6) revealed that the total knowledge rate in Yongle (83.4%) was significantly lower than that in Xinzhuang (95.5%). Over 90% of the respondents in both communities believed that schistosomiasis was a harmful disease endemic in Eryuan and knew that bovines can transmit schistosomiasis. Knowledge rates about the name, endemic areas, and way of transmission in Yongle were significantly lower than that in Xinzhuang. Only 1,394 (71.3%) respondents in Yongle linked the cause of schistosomiasis with coming into contact with infested water. Among respondents aged 15 years or below, more than one third didn’t know the name, endemic areas, and the animal reservoirs of schistosomiasis in both communities. In Yongle, 42.8% knew about the way of transmission, which was lower than that in Xinzhuang (59.8%), with a significant difference. Nearly 50% mistakenly wrote that coming into contact with sick people and eating undercooked fish were causes of the schistosomiasis infection. The source of schistosomiasis-related knowledge was investigated by multiple-choice questions. Most of the respondents in both communities acquired their knowledge from doctors, followed by handouts and hearing from others (see Figure 2).

**Table 3:** Respondents’ knowledge of, attitude towards, and practice relating to schistosomiasis

**Statement**	**Responses**	**Total**	**Yongle**	**Xinzhuang**	***χ***^**2**^	***P*-Value**
		**N = 2734**	**N = 1956**	**N = 778**		
K1.Do you know what schistosomiasis is?	Yes	90.2(61.6)	88.7(60.2)	94.2(66.4)	19.6	**<0.001**
	No	9.8(38.4)	11.3(39.8)	5.8(33.6)		
K2.Is schistosomiasis endemic in your area?	Correct	90.9(65.4)	89.7(65.6)	94.1(64.8)	13.2	**<0.001**
	Not correct	9.1(34.6)	10.3(34.4)	5.9(35.2)		
K3.How do people get infected by schistosomiasis?	Correct	76.8(46.6)	71.3(42.8)	90.9(59.8)	129.6	**<0.001**
	Not correct	23.2(53.4)	28.7(51.2)	9.1(40.2)		
K4.Do animals transmit schistosomiasis?	Correct	93.6(76.8)	92.9(76.2)	95.2(78.7)	7.5	**0.024**
	Not correct	6.4(23.2)	7.1(23.8)	4.8(21.3)		
K5.Which animals transmit schistosomiasis?	Bovines	93.6(76.4)	92.8(76)	95.8(77.9)	9.8	**0.043**
	Other	6.4(23.6)	7.2(24)	4.2(22.1)		
K6.Is schistosomiasis harmful?	Correct	95.7(83.7)	95(83.3)	97.4(85.2)	8.0	**0.005**
	Not correct	4.3(5.3)	5.0(9.7)	2.6(6.8)		
	Other	7.5(11)	8.7(14)	4.4(8)		
P1.Grazed (cattle or sheep) this year	No	85.4(94.7)	94.3(98.4)	63.1(82)	496.4	**<0.001**
	Occasionally^*^	8.0(4.2)	5.0(1.4)	15.7(13.9)		
	Frequently^*^	6.6(1.1)	0.8(0.2)	21.2(4.1)		
P2.Came into contact with water from rivers/ditches/field?	Yes	96.8(92.7)	97.5(95.1)	94.9(84.4)	12.9	**<0.001**
	No	3.2(7.3)	2.5(4.9)	5.1(15.6)		
P3.Left feces in open areas this year?	No	34(58.3)	35.4(59.5)	30.3(54.1)	32.6	**<0.001**
	Occasionally^*^	64.7(41.5)	64(40.5)	66.5(45.1)		
	Frequently^*^	1.4(0.2)	0.6(0)	3.2(0.8)		
P4.Dug vegetables and cut grass in the field this year?	No	22.3(0.2)	23.4(0)	19.5(64.7)	110.7	**<0.001**
	Occasionally^*^	31.1(58.5)	35.8(56.9)	19.0(27)		
	Frequently^*^	46.6(31.8)	40.7(33.2)	61.4(8.2)		
A1.Believes that schistosomiasis can be prevented	Yes	87.9(60.5)	86.4(61.6)	91.8(56.6)	15.1	**<0.001**
	No	12.1(39.5)	266(38.4)	64(43.4)		
A2.Willing to be checked up by doctors	Yes	99.2(97.3)	98.9(96.5)	100(100)	8.5	**0.004**
	No	0.8(2.7)	21(3.5)	0(0)		
A3.How livestock will be kept	Fencing	77.4(66.0)	76.4(64)	79.9(73)	15.1	**0.001**
	Free grazing	15.1(4.6)	14.9(3.8)	15.7(7.4)		
	Unknown	7.5(29.4)	8.7(32.2)	4.4(19.7)		

*“Occasionally” means less than five times per week and “frequently” means more than five times per week. *P*-value highlighted in bold means the difference was statistically significant. All values are percentages and data in “( )” refer to data of respondents aged.

Compared to the respondents from Yongle, those from Xinzhuang had more frequently grazed cattle or sheep, left feces in open areas, dug vegetables and cut grass in the field that year. Nevertheless, the percentage of respondents in Yongle (97.5%) who stated that they had a history of contact with water from rivers, ditches, or fields that year was higher than that in Xinzhuang (94.9%), with a significant difference. Further analysis found that among all respondents, half had come into contact with water due to farming (50%), followed by washing hands, swimming and playing, and washing clothes/dishes (see Table 4). Swimming and playing contributed as important risk factors for schistosomiasis in respondents who were aged 15 years or below, especially in the plateau basin areas (Yongle). Regarding the attitude towards schistosomiasis, 2,404 (87.9%) thought that schistosomiasis is a preventable disease, and nearly 100% were willing to be checked for it if doctors came to their village. The rates of having the right attitude towards schistosomiasis differ significantly between Yongle and Xinzhuang; 77.4% said they would continue to keep livestock by fencing (76.4% in Yongle versus 79.9% in Xinzhuang).

**Table 4:** Types of water contact among respondents in 2011

**Reason for water contact**	**Total N = 3357**	**Village**		***χ***^**2**^	***P*-Value**
		**Yongle**	**Xinzhuang**		
		**N = 2242**	**N = 1115**		
Swimming and playing	15.8	17.7	10.9	41.0	**<0.001**
Washing	1.9	2.3	0.9	9.3	**0.002**
Farming	60.6	54.5	76.1	9.2	**0.002**
Washing hands	44.4	40.1	55.4	4.4	**0.036**

*P*-Value highlighted in bold means the difference was statistically significant.

### The relationship between the knowledge score and the demographic data

The maximum knowledge score in our questionnaire is six and the minimum is zero. The mean knowledge score of respondents was 5.41. No disparities were detected among different genders or ethnicities. From Table 5 we can see that the odds of having a lower knowledge score was significantly higher in those respondents aged 15 years or below and among those who had a primary education level or below. In addition, respondents aged 16–30 years were more likely to get a lower knowledge score in Xinzhuang than in Yongle. Farmers and those aged between 31and 65 tended to have a higher knowledge score of schistosomiasis in both communities.

**Table 5:** Association between the respondents’ knowledge score and the demographic data

**Characteristics**	**Yongle (n = 1956)**		**OR, 95% CI**	**Xinzhuang (n = 778)**		**OR, 95% CI**
	**Scores ≤ 3, n (%)**	**Scores > 3, n (%)**		**Scores ≤ 3, n (%)**	**Scores > 3, n (%)**	
**Age group**						
5 – 15	155 (7.9)	270 (13.8)	17.4 [12.3,24.6]	40 (5.1)	82 (10.5)	22.3 [16.1,30.8]
16 – 30	21 (1.1)	397 (20.3)	0.4 [0.2,0.6]	0 (0)	111 (14.3)	79.5 [27.7,227.9]
31 – 45	13 (0.7)	637 (32.6)	0.1 [0.1,0.2]	3 (0.4)	285 (36.6)	0.1 [0.03,0.4]
46 – 65	13 (0.7)	434 (22.2)	0.2 [0.1,0.4]	1 (0.1)	246 (31.6)	0.04 [0.01,0.3]
≥66	2 (0.1)	14 (0.7)	1.2 [0.2,3.6]	0 (0)	10 (1.3)	-
**Education**						
Illiterate	14 (0.7)	193 (9.9)	0.6 [0.3,1.0]	3 (0.4)	124 (15.9)	0.4 [0.1,1.1]
Primary	145 (7.4)	798 (40.8)	2.9 [2.1,4.0]	37 (4.8)	357 (45.9)	5.6 [2.5,12.7]
Junior High	40 (2)	676 (34.6)	0.4 [0.3,0.6]	4 (0.5)	221 (28.4)	0.2 [0.08,0.7]
Senior High	4 (0.2)	76 (3.9)	0.4 [0.2,1.2]	0 (0)	26 (3.3)	0.4
College and above	1 (0.1)	9 (0.5)	0.9 [0.1,7.6]	0 (0.0)	6 (0.8)	1
**Occupation**						
Farming	50 (2.6)	1452 (74.2)	0.1 [0.04,0.09]	4 (0.5)	642 (82.5)	0.01 [0.01,0.04]
Student	141 (7.2)	285 (14.6)	11.5 [8.3,15.9]	39 (5)	84 (10.8)	60.4 [23.1,157.4]
Other	13 (0.7)	15 (0.8)	7.9 [3.7,16.8]	1 (0.1)	8 (1)	2.1 [0.3,17.3]

### The association between KAP and schistosomiasis seroprevalence

The respondents who live in Xinzhuang have 2.389 times the infection risk of those who live in Yongle (see Table 6). The respondents who grazed cattle or sheep frequently that year had a higher infection risk than those who grazed cattle or sheep occasionally. Respondents who dug vegetables or cut grass in the field frequently that year were 1.671 times more likely to get the infection than those who did not do this or did this occasionally. The odds for a schistosomiasis infection was 1.820 times higher in those individuals who worked with livestock by free grazing compared to those individuals who kept livestock by fencing. However, the seroprevalence of schistosomiasis was similar among respondents who came into contact with water from rivers, ditches and fields, and those who left feces in open areas compared to those who did not report any of these practices.

**Table 6:** Association of selected KAP related variables with schistosomiasis seroprevalence, Eryuan County, 2011

**Variables**		**Number**		***P*-Value**	**AOR [95% CI]**
		**Studied**	**Positive**		
Village	Yongle	1956	293	**<0.001**	1
	Xinzhuang	778	58	**<0.001**	2.389 [1.7,3.4]
Did you graze cattle or sheep?	No	1631	180	0.34	0.77 [0.5,1.3]
	Occasionally*	1078	168	**<0.001**	0.27 [0.1,0.6]
	Frequently*	25	3	**0.002**	1
Come in contact with water from the field?	Yes	351	345	NS	NS
	No	2383	2301	NS	
Left feces in open areas this year?	No	929	94	NS	NS
	Occasionally*	1768	252	NS	
	Frequently*	37	5	NS	
Dug vegetables or cut grass in the field?	No	604	56	**0.003**	1
	Occasionally*	849	110	0.361	1.179 [0.8,1.7]
	Frequently*	1275	184	**0.002**	1.671 [1.2,2.3]
What do you do with your livestock?	Fencing	2117	256	**<0.001**	1
	Free grazing	413	78	**<0.001**	1.820 [1.4,2.5]
	Unknown	204	17	0.231	0.724 [0.4,1.2]

AOR = adjusted odds ratio; NS = no significant.

*“Occasionally” means less than five times per week and “frequently” means more than five times per week.

Adjusted OR (adjusted odds ratio from multivariable logistic regression model) = when the effect of one factor on schistosomiasis prevalence is evaluated the analysis was adjusted for other remaining factors listed in the table.

## Discussion

The goals of the schistosomiasis strategic plan have been set by the World Health Organization (WHO) in 2012, indicating that the Western Pacific Region, including China, will interrupt schistosomiasis transmission by 2025 [22]. The Ministry of Health of P.R. China set the goal of interrupting the transmission of schistosomiasis in Yunnan by 2015, which means not acquiring anymore patients or infected livestock for five years following the achievement of transmission control in 2010 [6]. According to this study, the prevalence of schistosomiasis was 0.8%, which means one of the criteria of schistosomiasis control has been reached (<1% prevalence rate) [5]. Changing the ecological environment such as implementing the “Flourishing Forest and Controlling Snails Project” and sustained control strategies which include large-scale preventive chemotherapy, snail control, and control of the infection source may have contributed to this achievement [23].

For schistosomiasis control in the transmission areas of P.R. China, the Chinese health authority has set a goal that 80% of the population should have basic knowledge of schistosomiasis and its prevention methods [24]. In this study, the infection rate amongst farmers is higher than that among other occupations, though it was discovered that farmers have more knowledge about schistosomiasis. Analysis about the source of knowledge uncovered that respondents mainly found out about the disease from doctors, followed by handouts. We hypothesized that respondents found out about schistosomiasis from doctors after being infected with the disease. At the same time, misconceptions about the cause of schistosomiasis are still prevalent in Eryuan, as is having no knowledge about the disease, especially among children. This indicates that health education is critical for children to learn about the disease, and thus recognize and manage their health problems [25]. A review and meta-analysis on the effects of health education on schistosomiasis indicated that health education may be considerably important to prevent schistosomiasis, particularly after long-term implementation [12].

On the other hand, it was reported that children can act as behavior change agents in the community as their parents—who are likely to receive health education through their children—can be motivators and participate in household and community-based preventive actions [26]. Yunnan has reached the phase of transmission control for schistosomiasis in 2009, and is eligible for elimination [6,9]. Even though the infection situation of *S. japonicum* has reached a record low, further preventive and control measures are challenging to set and implement [6]. From the results of our survey, the infection rate was once highest in plateau basin areas (Yongle), but is now highest in plateau canyon areas (Xinzhuang) where the risk factors for schistosomiasis, such as frequently grazing cattle, digging vegetables, cutting grass in the field, and raising cattle by free grazing, were greater.

Moreover, this study revealed that coming into contact with water from rivers, ditches, and fields was not significantly associated with the seroprevalence of schistosomiasis. But this practice is key to being infected with schistosomiasis. Farming accounted for 50% of this risk behavior. Swimming and playing are important risk factors for schistosomiasis in respondents aged 15 years and below, especially in the plateau basin areas (Yongle). The in-depth interviews (IDIs) showed that farmers–due to their economic situation–did not wear long boots, meanwhile students were used to swimming in the natural pond rather than the swimming pool. Out of the respondents, 77.4% will continue to keep livestock by free grazing, even though this was found to contribute to higher infection rates. In a previous report, bovines were found to be an important source of infection in the Eryuan County [20]. However, the IDIs demonstrated that a complicated environment and tough economic conditions made it arduous to fence the livestock.

To avoid the risk factors summarized above, a more intensified strategy is required in areas where the aim is to interrupt transmission. Sustaining monitoring and control of the residual snails and treatment of the infection sources for schistosomiasis is a crucial strategy [3,10,27]. In addition, various control strategies should be designed keeping in mind the two different subtypes of the endemic areas for schistosomiasis (Yongle [plateau basins] and Xinzhuang [plateau canyons]). In both communities, especially in the plateau canyons, health education is a fundamental and sustainable way to raise community awareness about appropriate behaviors and preventive measures for schistosomiasis and other NTDs, especially in children aged 15 years and below. In addition, hand-washing facilities for farmers should be set up [28,29]. Previous studies showed that information on the cause and prevention of schistosomiasis could be introduced through multimedia (such as videos or TV programs) for students and famers to help improve and change risky behaviors [30,31]. It is suggested that ecological sanitary toilets should be improved. As well as that, economical crops which fit the environment of the plateau canyons are also needed to prevent people digging vegetables [32]. Meanwhile swimming pools for residents and tap water for washing clothes should managed, especially in plateau basins.

Knowledge, attitude, and practice (KAP) questionnaires are easy to use and collect data from individuals more reliably. But some related information may be missed because of the format of the questionnaire or the limited options. With respect to this limitation, IDIs were used in this study to collect further information. This study does have several limitations. Firstly, the study population may not fully represent all the people in low-endemic areas for schistosomiasis in Yunnan. This could be for several reasons including that many young people move to urban areas for work or study, thus leaving older people and children at home. In addition, a handful of health workers did not take part in the survey as they were too busy or traveling. Secondly, a qualitative survey which includes focus group discussions with community leaders, health facility personnel, and head teachers of the community schools are not included in this study, therefore details about the status of the health education in Eryuan are missing. Thirdly, in areas with low-level endemicity for schistosomiasis japonica, more sensitive diagnostic tools other than the Kato-Katz method for assessing intensities of *S. japonicum* infections are needed [33].

## Conclusion

According to this survey, the Eryuan County’s overall awareness of the cause and preventive measures of schistosomiasis was found to be high. However, knowledge gaps about the cause and transmission of schistosomiasis were also observed among students and farmers in both communities. There are more risk factors for schistosomiasis in plateau canyon regions than in plateau basin regions, which include frequently grazing cattle, digging vegetables and cutting grass in the field, as well as raising cattle by free grazing. Due to the various dominant risk factors, different control strategies should be designed keeping in mind the two different subtypes of the endemic areas for schistosomiasis in mountainous regions.

### Ethical considerations

The National Institute of Parasitic Diseases, Chinese Center for Disease Control and Prevention granted approval for this study. All adult subjects provided written informed consent and a parent or guardian provided written informed consent on behalf of all children participants.

## Competing interests

The authors declare that they have no competing interests.

## Authors’ contributions

LL conceived the study, carried out the data analysis, and drafted the manuscript. GJY mainly conceived the project and revised the manuscript. HRZ carried out the data analysis and revised the manuscript. KY and LA collected the data. XNZ revised the manuscript and provided intellectual input to the interpretation of the findings. All authors read and approved the final manuscript.

## Supplementary Material

Additional file 1 Multilingual abstracts in the six official working languages of the United Nations. Click here for file
