# Supplementary material for: Knowledge of, attitudes towards, and practice relating to schistosomiasis in two subtypes of a mountainous region of the People’s Republic of China
Source: Infect Dis Poverty. 2014 May 7;3:16. doi: 10.1186/2049-9957-3-16 (PMC4064289; doi:10.1186/2049-9957-3-16)
Supplement: Additional file 1 — Multilingual abstracts in the six official working languages of the United Nations. [file 2049-9957-3-16-S1.pdf]

Translation of the abstract into the six official working languages of the United Nations

مستوى الإدراك والمواقف والممارسات المتعلقة بمرض البلهارسيا في نمطين فرعيين من إحدى المناطق الجبلية بجمهورية الصين الشعبية

لو ليو، جو- جينج يانج، هونج- رو زهو، كون يانج، لين آي

نبذة

الخلفية: ما زال داء البلهارسيا اليابانية يمثل أحد الأمراض المستوطنة في نطاق خمسة أقاليم مرتبطة بمناطق المستنقعات والبحيرات بالإضافة إلى إقليمين يقعان في المناطق الجبلية بجمهورية الصين الشعبية. هذا وقد أظهرت الدراسات أن الإدراك الفردي والمجتمعي، والمواقف تجاه داء البلهارسيا، والعادات الصحية تمثل جميعها عوامل أساسية وحاسمة نحو منع داء البلهارسيا والقضاء عليه؛ وقد سعت هذه الدراسة لتقييم مدي إدراك، ومواقف وممارسات الأشخاص تجاه داء البلهارسيا في نمطين فرعيين من منطقة جبلية تقع في مقاطعة إيرويان، إقليم يوونان، بجمهورية الصين الشعبية. تهدف الدراسة إلي تقديم اقتراحات لوضع إجراءات ومعايير تحكم أكثر فاعلية وتحديداً فيما يتعلق بعملية انتقال المرض وكيفية منعه في نمطين فرعيين بمنطقة جبلية ذات معدلات عدوى منخفضة المستوى.

الطريقة: تم القيام بدراسة مقطعية عرضية تشمل عدد 3.000 من السكان الذين يقطنون بكل من منطقة يونجل (حوض الهضبة) و منطقة شينزهوانج (وادي الهضبة) بمقاطعة إيرويان، إقليم يوونان خلال شهري نوفمبر وديسمبر 2011؛ حيث تم أخذ عينات عشوائية عنقودية من طبقات مختلفة باستخدام مجموعة موحدة من استمارات الاستبيان الكمية التي يشرف عليها مساعدين مُدَربين؛ وبالإضافة إلي تقديم الدعم من خلال بيانات كيفية تم اختيارها بصورة عشوائية من مقابلات تفصيلية (IDIs) والتي تم إجراءها مع عشرة مزارعين وعشرة طلاب يقطنون في مناطق الدراسة المعنية. هذا وقد تم فحص جميع المشاركين فيما يتعلق بداء البلهارسيا باستخدام اختبار سيرولوجي (فحص تجمعات كرات الدم الحمراء (IHA) و فحص البراز (Kato-Katz).

النتيجة: أظهرت النتائج أن المعدل الكلي لمعرفة وإدراك داء البلهارسيا في منطقة يونجل كان (83.4%) بمعدل أقل بشكل ملحوظ عما كان موجود في منطقة شينزهوانج (95.5%)؛ وأنه في كلا المنطقتين، من بين المشاركين الذين يبلغون 15 عام من العمر أو أقل، كان هناك أكثر من ثلث المشاركين لا يعلمون شيئاً عن اسم، ومناطق توطن المرض، وأماكن التجمع الحيوانية للبلهارسيا؛ وأن أغلبية المشاركين بمنطقة إيرويان قد توصلوا لمعرفتهم بداء البلهارسيا من خلال الأطباء، ثم بعد ذلك من خلال المطبوعات والسماع من الآخرين؛ في حين كان المشاركين الذين يبلغون 15 عام من العمر أو أقل بالإضافة إلى السكان الحاصلين على مستوى تعليم أساسي يوجد لديهم معدل معرفة أقل بشكل أكثر احتمالاً في كلا المنطقتين. هذا وقد كان معدل العدوى هو الأعلى في أحد الاوقات من قبل في مناطق حوض الهضبة (يونجل)، ولكنه الآن هو الأعلى في مناطق وادي الهضبة (شينزهوانج)، حيث توجد عوامل أكثر خطورة بالنسبة لداء البلهارسيا، مثل الماشية التي ترعى هناك بشكل متكرر، زراعة الخضروات أو قطع الحشائش في الحقل، بالإضافة إلى تربية الماشية عن طريق الرعي الحر.

الخاتمة: وبإيجاز، فإن مستوى الوعي العام بمقاطعة إيرويان فيما يتعلق بالأسباب والإجراءات الوقائية لداء البلهارسيا قد اتضح أنه مرتفع، ولكن نتيجة للعوامل الخطيرة المختلفة التي تسود هناك، فإنه يجب وضع استراتيجيات تحكم تأخذ بعين الاعتبار بالنمطين الفرعيين المختلفين بمناطق توطن داء البلهارسيا في المناطق الجبلية، وهي أحواض الهضبة ووديان الهضبة.

Translated from English version into Arabic by Badran Hamed, through

TRANSLATORS

中国山丘型血吸虫病两种亚类疫区的血吸虫病相关知识、态度和行为现状

刘璐，杨国静，朱宏儒，杨坤，艾琳

摘要

引言：血吸虫病目前仍流行于中国的湖区 5 省和山区 2 省。以往的研究表明，个体及群体对血吸虫病的认知和态度以及他们的卫生行为，对血吸虫病的防治起到重要影响。本次研究选择了中国云南省洱源县山丘型血吸虫病两种亚类疫区，对其居民的血吸虫病相关知识、态度和行为的现状做了相关调查，为血吸虫病低流行山区的不同亚类疫区综合防治措施的制定提供依据和建议。

方法：本研究于 2011 年 11-12 月采用分层整群抽样方法，抽取了中国云南省洱源县永乐村（平坝型）和新庄村（峡谷型）3000 名 5 岁以上居民作为调查对象。经过培训的研究助理采用问卷调查的方法，并对随机抽取 10 名农民和 10 名学生进行深入访谈，从而调查居民的经济状况、血防知识、态度及相关行为。同时对所有调查对象采用间接血凝法和改良加藤法进行血吸虫病检测。

结果：永乐村（平坝型）血吸虫病总知晓率（83.4%）低于新庄村（峡谷型）（95.5%），差异有统计学意义。两个村庄中，均有超过三分之一的 15 岁以下的儿童不知道血吸虫病名称、流行区或者动物宿主；大多数的居民获得血吸虫病知识的来源为医生，其次为手册、从他人/处听说；15 岁以下年龄组和小学教育水平的人群血防知晓率最低。新庄的血吸虫病感染率由曾经低于到如今高于永乐村，与区域内存在多种血吸虫病危险因素有关，比如经常放牛、挖野菜割野草，以及对饲养牲畜采取散养的方式。

结论：洱源县血防意识普遍较高。但由于山丘型不同亚类疫区血吸虫病危险因素的不同，应根据山丘型血吸虫病不同亚类疫区的流行特征（平坝型和峡谷型），因地制宜制定血吸虫病综合防治措施。

Translated from English version into Chinese by Liu Lu, through

Connaissances, attitudes et pratiques concernant le schistosome chez deux sous-groupes de la population d'une région montagneuse de la République Populaire de Chine

Lu Liu, Guo-Jing Yang, Hong-Ru Zhu, Kun Yang, Lin Ai

Description

Contexte : Le Schistosomiasis japonica demeure endémique dans cinq provinces des régions lacustres et marécageuses et deux provinces des régions montagneuses de la République Populaire de Chine (R.P.C.). Des études ont démontré que la perception collective et individuelle, les attitudes envers la maladie et les mesures d'hygiène sont autant de facteurs cruciaux pour la prévention du schistosome. Cette étude a pour objectif de recenser les connaissances, attitudes et pratiques (KAP en anglais) concernant le schistosome de deux sous-groupes de la population de la région montagneuse du District d'Eryuan dans la Province de Yunnan en R.P. de Chine. Cette étude permettra de proposer la mise en place de mesures de contrôle plus ciblées et plus efficaces visant à réduire les risques de transmission de la maladie et à empêcher sa propagation dans les deux sous-groupes de la population de la région montagneuse qui ont un faible taux de contamination.

Méthode : Une étude transversale a été menée auprès de 3 000 habitants dans les communautés de Yongle (bassin) et Xinzhuang (canyon) du District d'Eryuan de la Province de Yunnan entre novembre et décembre 2011. Un échantillonnage aléatoire stratifié en grappes a été réalisé à l'aide d'un ensemble uniforme de questionnaires quantitatifs complétés par des sondeurs qualifiés. Cet échantillonnage est accompagné de données qualitatives recueillies de manière aléatoire auprès de dix fermiers et dix étudiants de la région concernée au cours d'entretiens approfondis. Tous les participants ont passé des examens à la recherche du schistosome : analyse sérologique (réaction d'hémagglutination indirecte) et analyse des selles (Kato-Katz).

Résultat : Au total, seul 83,4% de la population des Yongle connaissaient l'affection contre 95,5% des Xinzhuang. Dans les deux communautés, parmi les personnes interrogées âgées de moins de 15 ans, plus d'un tiers ne connaissaient ni le nom de la maladie, ni les régions endémiques, ni les espèces-réservoirs du schistosome. La majorité des personnes interrogées dans le District d'Eryuan avait appris la connaissance du schistosome soit par les médecins, soit en lisant une brochure, soit via le bouche-à-oreille. Les personnes interrogées de moins de 15 ans ou ayant un

faible niveau d'éducation avaient une connaissance générale de la maladie moins développée dans les deux communautés. Le taux d'infection était auparavant plus élevé dans les régions du bassin (Yongle) mais aujourd'hui, ce sont les régions du canyon qui sont les plus touchées, avec un plus grand nombre de facteurs de risques tels que la présence de pâturages à bétail, le travail de la terre ou le fauchage de l'herbe ainsi que l'élevage du bétail en plein air.

Conclusion : Pour conclure, les habitants du District d'Eryuan sont généralement plutôt bien informés sur les causes et les mesures de prévention du schistosome. Cependant, en raison des nombreux facteurs de risques dominants, les différentes stratégies de contrôle devront être élaborées en tenant compte des deux sous-groupes de population des régions endémiques du schistosome dans les régions montagneuses, à savoir les bassins et les canyons.

Translated from English version into French by Fanny, through

TRANSLATORS
WITHOUT BORDERS

Осведомленность, отношение и врачебная практика применительно к шистосомозу в двух подтипах горных районов Китайской Народной Республики

Ли Лю, Го-Цзин Ян, Хун-Жу Чжу, Кунь Ян, Линь Ай

Аннотация

Предыстория: Японский шистосомоз по-прежнему распространен в пяти провинциях с озерно-болотистой местностью и двух провинциях с горной местностью в Китайской Народной Республике (КНР). Исследования выявили, что для предотвращения шистосомоза важны знания каждого отдельного человека и сообщества в целом, отношение к шистосомозу и гигиена. Наблюдения проводились, чтобы оценить осведомленность, отношение и врачебную практику применительно к шистосомозу в двух подтипах горных районов уезда Эрюань, провинции Юньнань, КНР. Цель обследования – внести предложения по более точным и действенным мерам борьбы с передачей и распространением заболевания в двух подтипах горных районов с низкой степенью заражения.

Метод: В ноябре и декабре 2011 года в Юнлэ (во впадине плоской возвышенности) и Синьчжуане (в ущелье плоскогорья) уезда Эрюань, провинции Юньнань, проводилось единовременное обследование 3000 жителей. Случайно выбранные представители разных слоев населения отвечали на ряд одних и тех же вопросов под руководством обученных специалистов. Полученные сведения впоследствии были подкреплены качественными данными, случайным образом отобранными из подробных бесед с десятью фермерами и десятью студентами из исследуемых районов. Все участники проверялись на шистосомоз с помощью серологического анализа (реакции непрямой гемагглютинации) и исследования кала (по методу Като-Каца). **Результат:** Общий процент осведомленности о шистосомозе в Юнлэ (83,4%) оказался значительно ниже, чем в Синьчжуане (95,5%). В обоих населенных пунктах более трети опрашиваемых в возрасте не старше 15 лет не знали названия, областей распространения и животных, которые являются возбудителями шистосомоза. Большинство опрашиваемых в Эрюане узнали о шистосомозе от врачей, других людей и из раздаточных материалов. В обоих населенных пунктах опрашиваемые не старше 15 лет и жители с начальным

образованием чаще обладали более низким уровнем знаний. Когда-то степень заражения была самой высокой в районах впадины (Юнлэ), но теперь болезнь чаще всего распространяется в районах ущелья (Синьчжуан), где больше факторов риска развития шистосомоза из-за регулярного выгона крупнорогатого скота, земляных работ, кошения травы в поле, а также разведения крупнорогатого скота на вольной пастьбе.

Вывод: Словом, общий уровень осведомленности о причинах и мерах предотвращения шистосомоза в уезде Эрюань считается высоким. Но из-за различных доминирующих факторов должны быть разработаны разные методы контроля для двух различных подтипов районов распространения шистосомоза в горных местностях, а именно для жителей впадин и ущельев плоскогорья.

Translated from English version into Russian by Zoya Nayshtut, through

TRANSLATORS
WITHOUT BORDERS

Conocimiento, actitud y prácticas relacionadas con la esquistosomiasis de dos subtipos en una región montañosa de la República Popular China

Lu Liu, Guo-Jing Yang, Hong-Ru Zhu, Kun Yang, Lin Ai

Resumen

Información de referencia: La esquistosomiasis japónica aún es endémica en la República Popular China, concretamente en cinco provincias de regiones con lagos y marismas y en dos provincias de regiones montañosas. Los estudios han indicado que la percepción individual y de la comunidad, las actitudes hacia la esquistosomiasis y los hábitos higiénicos son factores críticos para prevenir la esquistosomiasis. Este estudio trataba de evaluar el conocimiento, la actitud y las prácticas relacionadas con la esquistosomiasis de dos subtipos en una región montañosa en situada en la región de Eryuan (provincia de Yunnan). El objetivo del estudio es aconsejar la puesta en práctica de medidas de control más específicas y eficaces para la transmisión e interrupción de dos subtipos de la enfermedad en una región montañosa con tasas de infección de bajo nivel.

Método: Se llevó a cabo un estudio transversal de 3.000 habitantes en las comunidades de Yongle (cuenca mesetaria) y Xinzhuang (cañón mesetario) en la región de Eryuan (provincia de Yunnan) en noviembre y diciembre de 2011. Se realizó un muestreo aleatorio estratificado por grupos mediante un conjunto uniforme de cuestionarios cuantitativos administrados por ayudantes previamente formados. Se contó además con el soporte de datos cualitativos seleccionados de manera aleatoria de las entrevistas en profundidad realizadas con diez campesinos y diez estudiantes de las regiones estudiadas. Se examine a todos los participantes por esquistosomiasis mediante una prueba serológica (ensayo de hemaglutinación indirecta [IHA]) y un examen de deposiciones (Kato-Katz).

Resultado: La tasa total de conocimiento de la esquistosomiasis en Yongle (83,4%) era significativamente más reducida que en Xinzhuang (95,5%). En ambas comunidades, entre los encuestados de 15 años de edad o menos, más de un tercio no conocía el nombre, las zonas endémicas y los animales que transmiten la esquistosomiasis. La mayoría de los encuestados en Eryuan conocían la esquistosomiasis a través de los médicos, seguidos por los folletos informativos y por oírlo de otras personas. Los encuestados de 15 años de edad o menos y los residentes con nivel de educación primaria eran más proclives a tener un menor nivel de

conocimiento en ambas comunidades. La tasa de infección llegó a ser la más elevada en las zonas de la cuenca mesetaria (Yongle) pero ahora se da en las zonas del cañón mesetario (Xinzhuang), donde existen más factores de riesgo para la esquistosomiasis, como el ganado que a menudo pasta libremente, el cultivo de verduras o el corte de hierbas en el campo, así como la cría de ganado que pasta libremente.

Conclusión: El resumen, se averiguó que el conocimiento general en la región de Eryuan de las causas y las medidas para la prevención de la esquistosomiasis era elevado. Pero debido a los diversos factores de riesgo dominantes deberían diseñarse diferentes estrategias de control teniendo en cuenta los dos subtipos distintos de zonas endémicas para la esquistosomiasis en regiones montañosas, como son las cuencas mesetarias y los cañones mesetarios.

Translated from English version into Spanish by Sergiman, through

TRANSLATORS
WITHOUT BORDERS
